# Botulinum Toxin Type A for Preventing Facial Trauma and Hypertrophic Scars: A Meta‐Analysis and Trial Sequential Analysis

**DOI:** 10.1111/jocd.70501

**Published:** 2025-10-28

**Authors:** Jie Lu, Yuhua Chen, Hongxia Xie, Yiming Wang

**Affiliations:** ^1^ Medical Cosmetic Center Chengdu Second People's Hospital Chengdu China; ^2^ Department of Dermatology The Second People's Hospital of Chengdu China

**Keywords:** meta analysis, scar, sequential analysis of experiments, system evaluation, type A botulinum toxin

## Abstract

**Objective:**

To evaluate the effectiveness and safety of local injection of botulinum toxin type A in preventing hypertrophic scars after facial trauma and surgery using meta‐analysis and sequential experimental analysis methods.

**Method:**

Computer retrieval of randomized controlled trials on the prevention of facial scars with botulinum toxin type A from PubMed, EMbase, the Cochrane Library, CNKI, China Biomedical Literature Service System, Wanfang Database, and VIP Chinese Science and Technology Journal Full text Database up to February 2025.

**Result:**

Twelve randomized controlled clinical trials were included, with a total of 644 patients; Type A botulinum toxin injection is superior to the control group in improving patient satisfaction [*RR =* 6.89; 95% CI (3.20, 14.85); *p <* 0.0001], Vancouver Scale score [*RR =* −1.40; 95% CI (−2.73, 0.08); *p <* 0.0001], visual analog score [*RR =* 1.41; 95% CI (0.26, 2.56); *p =* 0.02], scar width [*RR =* −0.14; 95% CI (−0.17, −0.10); *p <* 0.0001], and the difference is significant; The incidence of adverse events [*RR =* 0.38; 95% CI (0.18, 0.84); *p =* 0.02] and recurrence rate [*RR =* 0.17; 95% CI (0.04, 0.81); *p <* 0.03] were lower than those in the control group, and no serious adverse reactions occurred; Sensitivity analysis showed that the results were relatively robust, sequential analysis of the experiment showed that the benefits were conclusive, and Begg's and Egger's tests showed no publication bias.

**Conclusion:**

Type A botulinum toxin injection has a certain therapeutic effect on hypertrophic scars without significant side effects. However, the accuracy and stability of its therapeutic effect still require more high‐quality research verification.

## 1 Introduction

Hypertrophic scar (HS) is a pathological repair product of wound fibrosis characterized by excessive collagen deposition that elevates tissue above the skin surface. It compromises appearance, restricts joint motion, hampers facial expression, and may impair organ function, imposing a serious psychosocial burden [1, 2]. HS in the maxillofacial and neck regions remains a global concern [3]. Lesions proliferate rapidly within 3–6 months after wound closure and then gradually regress, causing erythema, tension, and pruritus [4]. The mechanism is incompletely understood [5]; however, accumulating evidence indicates that mechanical tension around the wound is the most critical determinant of HS: the higher the pressure, the greater the risk [6, 7]. In vitro, excessive tension inhibits fibroblast apoptosis, promotes migration, and drives fibrosis [8]. Current management includes compression, laser, pharmacotherapy, surgery, cryotherapy, and radiotherapy [9], yet early intervention remains controversial, especially for facial and cervical wounds, because the biology of excessive scarring is poorly elucidated.

Botulinum toxin type A (BTX‐A), a neurotoxin produced by *Clostridium botulinum* , blocks presynaptic acetylcholine release at the neuromuscular junction by cleaving SNAP‐25 protein [10, 11]. Eight serotypes (A–G) are known [11]. First documented in 1897 and approved in the USA in 1989, BTX‐A has been used in China since 1997 [12, 13]. Plastic‐surgery applications have expanded to include rhytides, masseteric hypertrophy, hyperhidrosis, wound healing, and pathological scars such as keloids and hypertrophic scars [14]. In 1999, Gassner et al. reported the first randomized, double‐blind, placebo‐controlled primate study showing that peri‐incisional BTX‐A attenuates scar hypertrophy through reduction of wound tension and modulation of fibroblast activity [15]. Subsequent clinical trials have replicated these benefits with high patient satisfaction and minimal adverse events [16].

Despite growing evidence, the efficacy of BTX‐A for preventing postoperative HS remains contentious because individual trials are underpowered and large multicenter studies are costly [17]. Systematic reviews incorporating homogeneous randomized controlled trials (RCTs) can overcome these limitations through pooled analysis, while trial sequential analysis (TSA) further safeguards against random error and false‐positive conclusions in meta‐analyses [18]. Only a handful of systematic reviews have examined BTX‐A for HS prevention. These syntheses consistently report favorable improvements in Vancouver Scar Scale scores and patient satisfaction, yet they share common limitations: small pooled samples, absence of trial‐sequential analysis, heterogeneous injection protocols, and lack of face‐neck subgroup data. A dedicated systematic review with TSA focusing specifically on facial HS is therefore warranted to overcome these limitations and provide more robust clinical guidance regarding optimal BTX‐A dosing, timing, and injection techniques for maxillofacial scar prevention.

## 2 Materials and Methods

### 2.1 Literature Search

Search PubMed, Cochrane Library, Embase database, China National Knowledge Interest (CNKI), Wanfang Medical Database, VIP database from database inception to December 2024, and the Chinese search terms are “botulinum toxin type A” or “botulinum toxin” and “scar tissue”. Taking PubMed's search formula as an example, the English search includes “keloid” [MeSH Terms] OR “keloid” [All Fields] AND (“botulinum toxins” [MeSH Terms] OR (“botulinum” [All Fields] AND “toxins” [All Fields]) OR “botulinum toxins” [All Fields] OR (“botulinum” [All Fields] AND “toxins” [All Fields]) OR “botulinum toxins” [All Fields]). In addition, extensive data collection was conducted through methods such as literature review and manual searches, including those established by Cochrane Collaboration, randomized controlled trial registration system, conference papers, unpublished dissertations, etc. The search strategies for the main databases are shown in Table 1.

**TABLE 1:** The search strategies for the main databases.

Data base	Search mode (Bilingual)	Restrictions	Number of articles detected (primary inspection)	Remarks
PubMed	((“keloid”[MeSH Terms] OR “keloid”[All Fields] OR “hypertrophic scar”[All Fields] OR “scar”[All Fields]) AND (“botulinum toxins, type A”[MeSH Terms] OR “botulinum toxin type A”[All Fields] OR “BTX‐A”[All Fields] OR “onabotulinumtoxinA”[All Fields] OR “Botox”[All Fields])) AND (Randomized Controlled Trial[ptyp])	Published from January 1, 2000 to February 28, 2025; Languages: English/Chinese; Human Research	124	MeSH+ free‐text dual‐track; use RCT, filter
EMbase	(‘keloid’/exp. OR ‘hypertrophic scar’/exp. OR scar) AND (‘botulinum toxin A’/exp. OR ‘onabotulinumtoxinA’ OR ‘botox’ OR ‘BTX‐A’) AND [randomized controlled trial]/lim	2000–2025; Human; English/Chinese	137	Embase subject terms +EMTREE match
Cochrane Library (CENTRAL)	(“keloid” OR “hypertrophic scar” OR “scar”) AND (“botulinum toxin type A” OR “BTX‐A” OR “onabotulinumtoxinA” OR “Botox”)	Publication year 2000–2025	28	CENTRAL already has the RCT tag built in
CNKI	SU = ‘A type botulinum toxin’ + ‘botulinum toxin’ AND SU = ‘scar’ + ‘keloid’ AND FT = ‘random’	Publication date: January 1, 2000 to February 28, 2025; Journal/PhD/Conference	97	SU = Subject; FT = Full text
Wanfang Medical Network	Subject: (Type A Botulinum toxin OR botulinum toxin) AND Subject: (scar OR keloid) AND Title or keywords: (randomized OR controlled)	2000–2025; medical journals/theses/conferences	83	Select “core journals” to reduce the number to 37
VIP	U = (A‐type botulinum toxin + botulinum toxin) (scar + keloid) R = random	2000–2025; all journals	65	U = any field; R = title/keyword
CBM (SinoMed)	“Cytoxin‐type/Bacillus botulinum type A toxin” [unweighted: expanded] AND (“scar” [unweighted: expanded] OR “keloid” [unweighted: expanded]) AND “randomized controlled trial” [unweighted: expanded]	2000–2025; Human; Chinese	101	Combination of CBM subject terms and subtopics

### 2.2 Inclusion and Exclusion Criteria

#### 2.2.1 Inclusion Criteria

Population (P): Adults aged 18–65 years of either sex who have developed post‐traumatic hypertrophic scars on the face or neck within the previous 6 months, confirmed by Vancouver Scar Scale (VSS) ≥ 6.

Intervention (I): Single intralesional or peri‐incisional injection of botulinum toxin type A (onabotulinumtoxinA, abobotulinumtoxinA, or incobotulinumtoxinA) as the sole experimental treatment, total dose 5–20 U or 2.5–5 U per linear cm, administered ≤ 14 days after wound closure or primary surgery.

Comparator (C): Placebo injection (normal saline), blank control (no injection), or standard monotherapy such as single intralesional triamcinolone.

Outcomes (O): Scar thickness (ultrasound/histology) and Vancouver Scar Scale total score at 3 and 6 months; secondary outcomes include scar width, POSAS, VAS for symptoms, and adverse events.

Study design (S): Randomized controlled trials (RCTs) with parallel or split‐scar design, sample ≥ 20 patients per arm, published in English, with ethics approval and extractable means ± SD or 95% CI.

#### 2.2.2 Exclusion Criteria

Population (P): Individuals < 18 or > 65 years; pregnant or lactating women; active local skin infection; neuromuscular disorders (e.g., myasthenia gravis, Eaton‐Lambert syndrome); known hypersensitivity to botulinum toxin; prior BTX‐A injection in the target area within 6 months; keloids, burn scars, or patients unable to complete follow‐up.

Intervention (I): Studies combining BTX‐A with any other active scar therapy (laser, steroids, 5‐FU, silicone, pressure garments, etc.) or those using repeat BTX‐A injections.

Comparator (C): Arms containing multiple simultaneous active treatments.

Outcomes (O): Trials lacking quantitative data sufficient to calculate weighted mean difference and 95% CI.

Study design (S): Non‐randomized studies, quasi‐experimental designs, animal experiments, case reports, letters, editorials, conference abstracts, or studies without documented ethics approval.

### 2.3 Literature Screening and Data Extraction

Two researchers independently conducted literature searches according to the search strategy, screened literature based on research objectives, inclusion and exclusion criteria, and made choices. They introduced a third reviewer in case of disagreement. They used the Cochrane risk bias assessment tool to evaluate the quality of literature and extracted data based on a pre‐designed data extraction table. Finally, cross‐checking is conducted. If there is a disagreement on the inclusion of literature, quality evaluation, and data extraction, it can be resolved through discussion, or a third‐party personnel can be invited to jointly discuss and evaluate. If encountering research with unclear or insufficient information, they try to contact the original author to confirm and supplement. The extracted content includes: (1) Basic information for inclusion in the literature: title, first author, publication date, etc.; (2) The total number of research subjects, treatment plans, and the sample size of each plan group; (3) Factors related to bias risk assessment.

### 2.4 Literature Quality Evaluation

Conduct bias risk assessment on the included RCT studies using the Cochrane bias risk assessment tool, which includes: (1) Is the method for generating random allocation sequences correct? (2) Whether the hidden means of the allocation plan are complete; (3) Is there any bias caused by the lack of blinding for researchers and research subjects? (4) Is there a measurement bias in the results due to the evaluator's knowledge of the grouping and intervention measures? (5) Whether the result data is complete and whether there is bias due to the quantity, nature, and processing methods of incomplete data; (6) Is there any bias caused by selective reporting of results? (7) Bias related to the adopted design or other issues. The evaluator uses the risk assessment criteria of “low bias risk”, “high bias risk”, and “uncertain bias risk” to evaluate each research item and uses Revman5.3 software to create a bias risk assessment chart based on the evaluation results.

### 2.5 Outcome Measures

The final outcome measures of the study can be divided into six parts. Primary outcomes: patient satisfaction, Vancouver Scale score (VSS); Secondary outcomes:

Visual Analog Scale (VAS), scar width, recurrence rate, and adverse reactions. All observation indicators are independently recorded by experienced observers, and after the observation indicators are determined, statistical software is used for statistical processing.

### 2.6 Statistical Methods

Meta analysis was conducted using RevMan 5.3 statistical software provided by the Cochrane Collaboration. Relative risk (RR) was used as the statistical measure for efficacy analysis in the count data; the measurement data adopts weighted mean deviation (WMD) or standard mean deviation (SMD), and each effect measure is represented by 95% CI. When there is no statistical heterogeneity between studies (*p* > 0.05, *I* ^2^ < 50%), a fixed effects model (M‐H, Fixed) is used for meta‐analysis of each study; if there is statistical heterogeneity between studies (*p <* 0.05, *I* ^2^ > 50%), a random effects model (M‐H, Random) will be used for analysis, and subgroup analysis will be conducted based on possible heterogeneity factors to test the heterogeneity between studies. TSA 0.9 software is used for sequential analysis of experiments. If the *Z*‐value curve reaches the threshold of sequential analysis, it indicates that the observed results of the current information content are conclusive. Use STATA 12.0 software to perform Egger linear regression to determine the presence of publication bias and conduct sensitivity analysis to exclude individual studies.

## 3 Results

### 3.1 Basic Characteristics of Search Results and Included Studies

**FIGURE 1:**
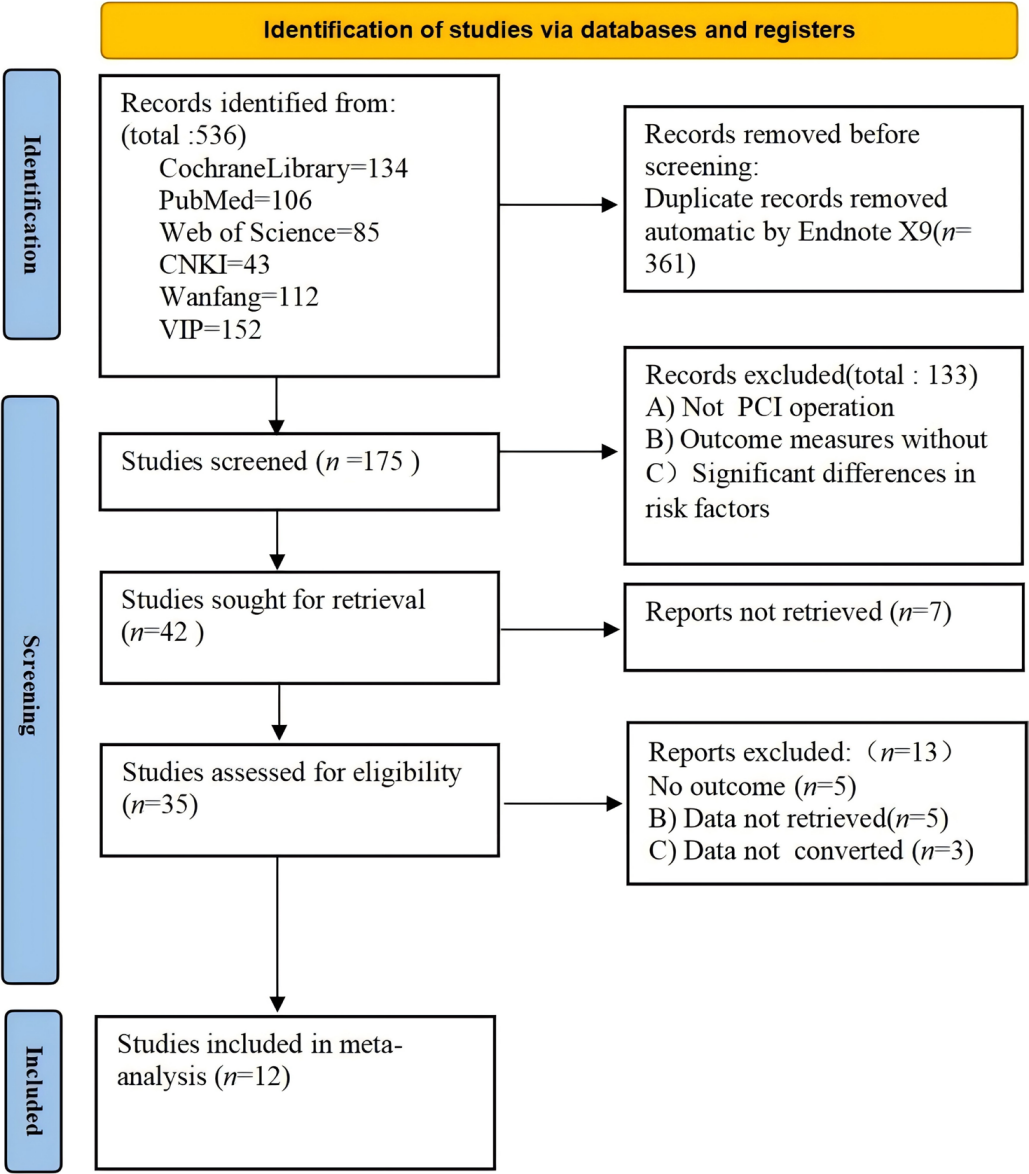
Literature screening process diagram.

536 articles were obtained through initial screening, and 6 articles were obtained through follow‐up reference supplementation. (1) Exclude literature that is duplicated in different databases (361 articles); (2) Read the title and abstract to exclude literature (133 articles); (3) Read the full text, screen, and exclude case reports, clinical symptom analysis, reviews, and abstracts of conference content that cannot be obtained in full according to inclusion criteria (34 articles); (4) Finally, 12 articles were included, with 644 patients included. Basic information is shown in Table 2. The literature screening process is shown in Figure 1, and the basic information and baseline characteristics of the included studies are shown in Table 1.

**TABLE 2:** Basic information of included data.

Lead author	Number of cases	Age	Male proportion	Intervention measures		Injection method	Observation time (month)	Outcome indicators
				Test group	Control group			
Chang2014 [19]	30/29	45/48	58/60	Botulinum toxin A	Normal saline	Postoperative dose: 1 U/kg	6	②③④
Chang2014 [20]	30/38	46/46	50/53	Botulinum toxin A	Normal saline	Postoperative dose: 1 U/cm	6	②③④
Huang2023 [21]	49/48	42/38	51/54	Botulinum toxin A	Normal saline	Postoperative, 1 U/injection point	6	②③⑤⑥
Hu2018 [22]	19/19	37.3/38.2	52.7/51.9	Botulinum toxin A	Normal saline	Postoperative, 1 U/injection point	12	②③④
LEE2018 [23]	15/15	47.5/44.9	56.9/56.8	Botulinum toxin A	None	5 days after surgery	6	②④
Liu2023 [24]	50/50	32/32	59.8/50.7	Botulinum toxin A	Normal saline	72 h after surgery, 1 U/injection point	6	③⑥
Tao2018 [25]	18/18	—	—	Botulinum toxin A	None	Postoperative, 1.5 U/cm	12	①⑤
Wang2011 [26]	30/30	38/36	56/53	Botulinum toxin A	Normal saline	72 h after surgery, 5 U/injection point	12	①
Wang2015 [27]	35/35	42/39	53/51	Botulinum toxin A	Normal saline	72 h after surgery, 1 U/injection point	6	①②④
Xu2019 [28]	18/18	49/49	57/55	Botulinum toxin A	None	After surgery, 10 U/cm	6	①②③④
ZELKEN2016 [29]	13/13	40/40	51/53	Botulinum toxin A	Normal saline	10 days after surgery, 2 U/injection point	27	③
ZIADE2013 [30]	11/13	51.4/52.6	56/59	Botulinum toxin A	Normal saline	72 h after surgery, the dosage will be determined by the doctor	12	①③

*Note:* ① Patient satisfaction, ② Vancouver Scale Score (VSS), ③ Visual Analog Scale (VAS), ④ Scar width, ⑤Recurrence rate, ⑥Adverse reactions.

### 3.2 Quality Evaluation

**FIGURE 2:**
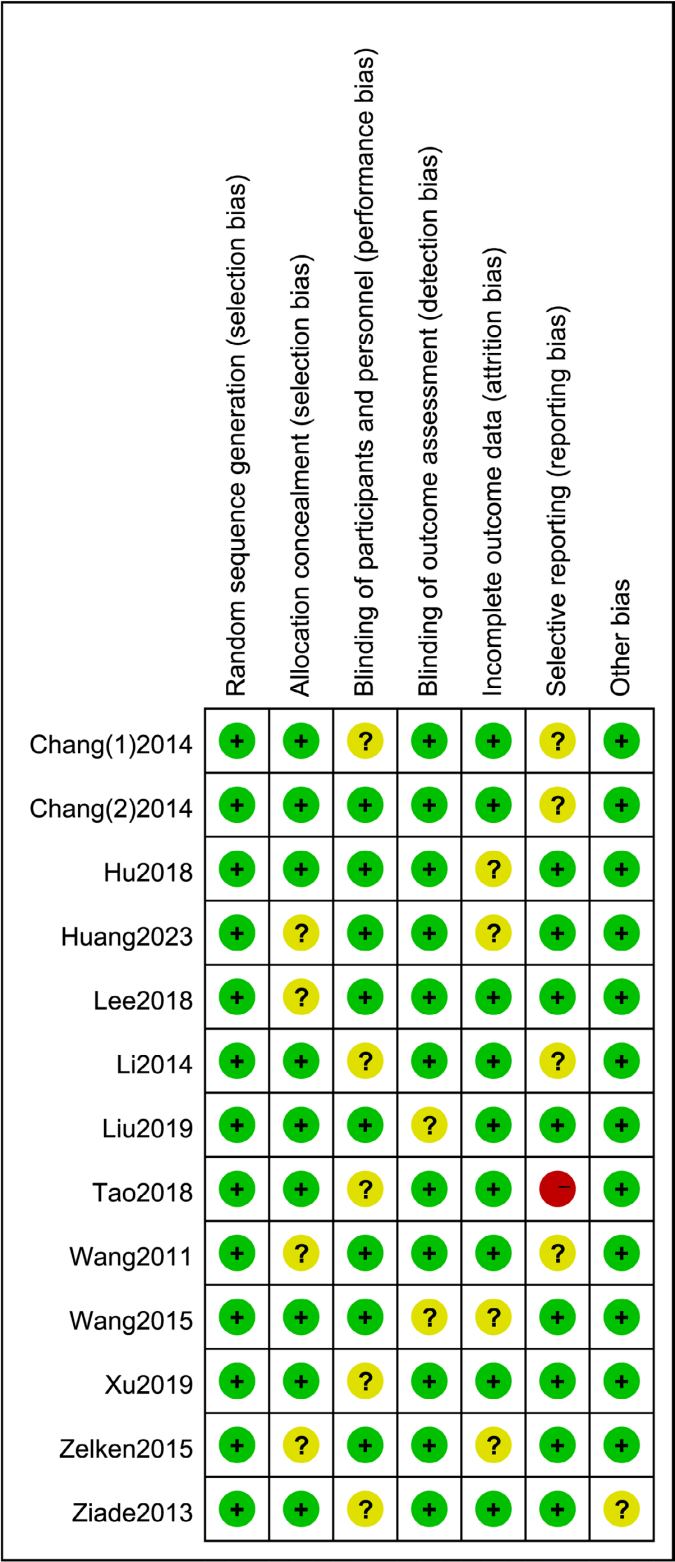
Risk of bias graph.

**FIGURE 3:**
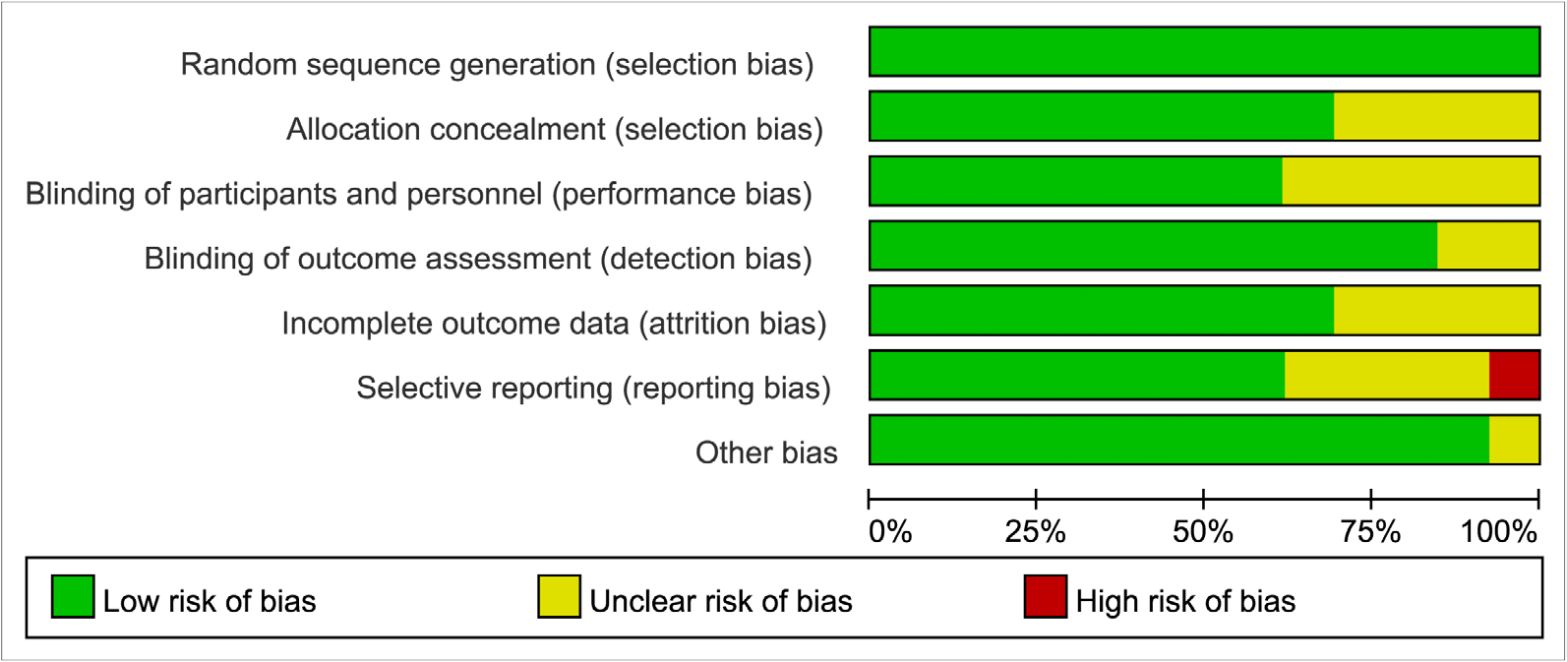
Risk of bias summary.

Methodological quality evaluation of included studies, four included studies did not mention allocation scheme concealment, five included studies did not mention outcome measurement blinding, and two included studies did not mention selective reporting; the four included studies did not mention or describe whether blinding was used for judgment. One study indicated that two individuals were lost to follow‐up during the trial and were classified as “high risk”; All studies indicate that the baseline conditions of the two groups are similar. The methodological quality evaluation included in the study is detailed in Figures 2 and 3.

### 3.3 Statistical Analysis Results

#### 3.3.1 Patient Satisfaction

**FIGURE 4:**
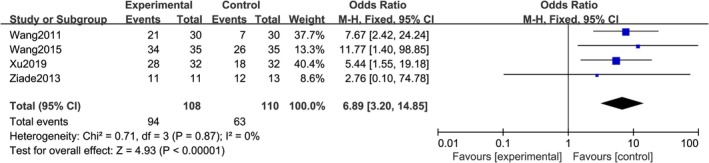
Forest chart of patient satisfaction.

A total of four studies reported patient satisfaction, with no statistical heterogeneity between the studies (*I* ^2^ = 0%, *p =* 0.87), using a fixed effects model. The meta‐analysis results showed a statistically significant difference in patient satisfaction between the botulinum toxin group and the control group [*RR =* 6.89; 95% CI (3.20, 14.85); *p <* 0.0001]. Injecting botulinum toxin A around the wound can reduce discomfort and improve patient satisfaction (Figure 4).

#### 3.3.2 Vancouver Scale Score (VSS)

**FIGURE 5:**
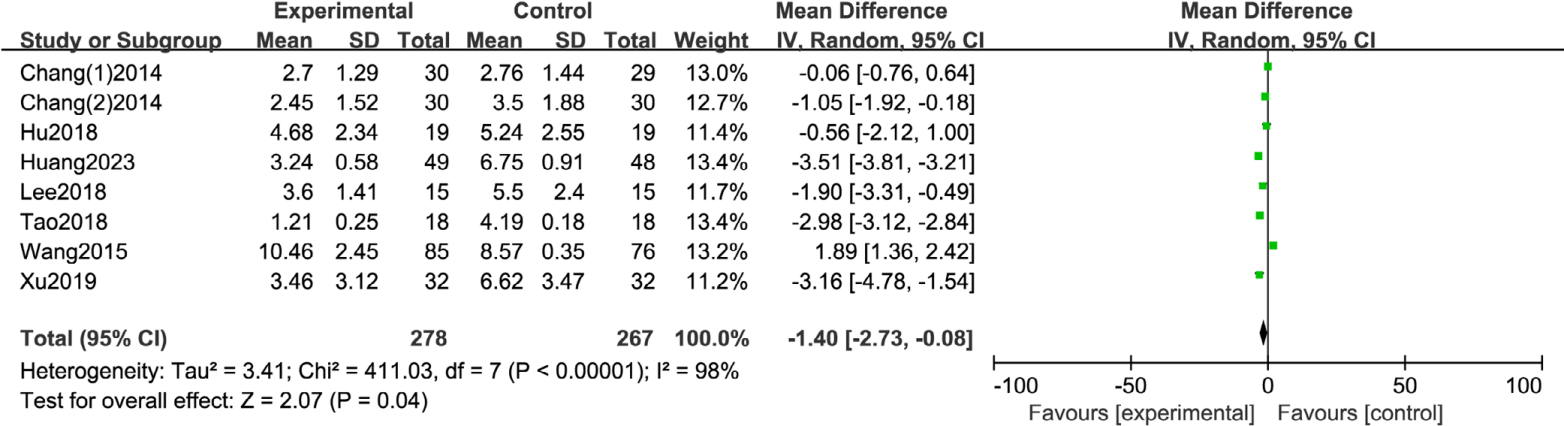
VSS rating forest chart.

A total of 8 studies reported on VSS, with statistical heterogeneity between each study (*I* ^2^ = 0%, *p =* 0.87), using a random effects model. The meta‐analysis results showed a statistically significant difference in VSS scores between the two groups [*RR =* −1.40; 95% CI (−2.73, 0.08); *p <* 0.0001] (Figure 5).

#### 3.3.3 Visual Analog Scale (VAS)

**FIGURE 6:**
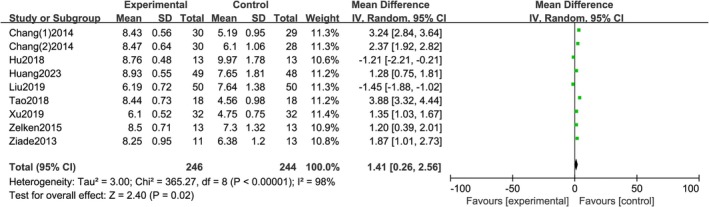
Forest plot of VAS score.

A total of 9 studies reported VAS, with statistical heterogeneity between each study (*I* ^2^ = 100%, *p <* 0.0001), using a random effects model. The meta‐analysis results showed a statistically significant difference in VAS scores between the two groups [*RR =* 1.41; 95% CI (0.26, 2.56); *p =* 0.02]. The results indicate that local injection of botulinum toxin A in the early stage of wound healing can significantly improve scar quality, as shown in Figure 6.

#### 3.3.4 Scar Width

**FIGURE 7:**
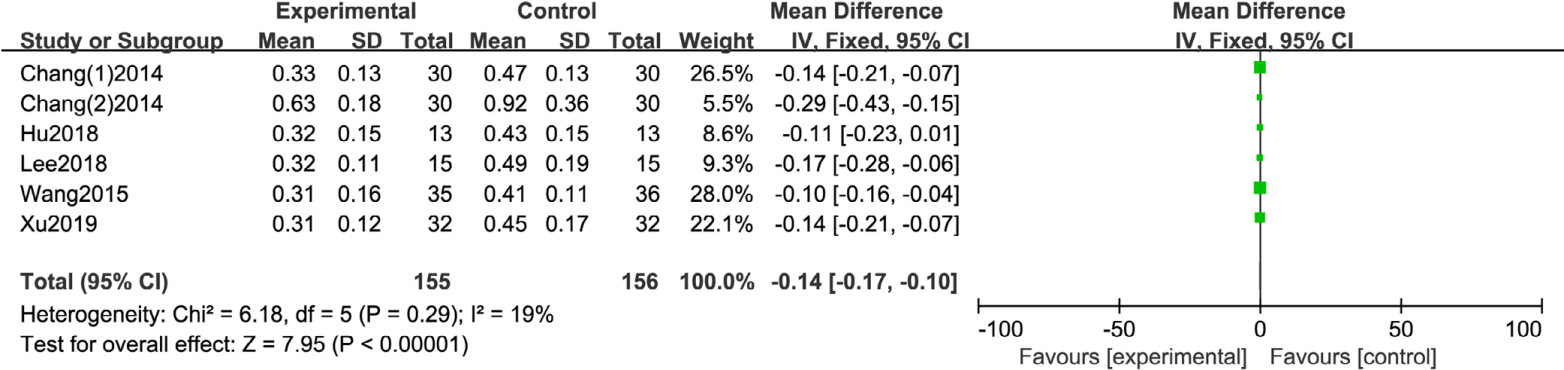
Scar width forest map.

A total of 6 studies reported VAS, with no statistical heterogeneity between studies (*I* ^2^ = 19%, *p =* 0.29), using a fixed effects model. The meta‐analysis results showed a statistically significant difference in scar width between the two groups [*RR =* −0.14; 95% CI (−0.17, −0.10); *p <* 0.0001]. The results indicate that injecting botulinum toxin A around the wound can reduce the width of facial and neck scars, as shown in Figure 7.

#### 3.3.5 Recurrence Rate

**FIGURE 8:**
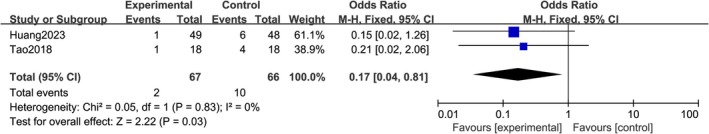
Forest plot of recurrence rate.

Two studies reported recurrence rates, with no statistical heterogeneity between each study (*I* ^2^ = 0%, *p =* 0.83), and a random effects model was used. The meta‐analysis results showed a statistically significant recurrence rate between the two groups [*RR =* 0.17; 95% CI (0.04, 0.81); *p <* 0.03]. The results indicate that injecting botulinum toxin A around the wound can reduce the recurrence rate, as shown in Figure 8.

#### 3.3.6 Adverse Reactions

**FIGURE 9:**
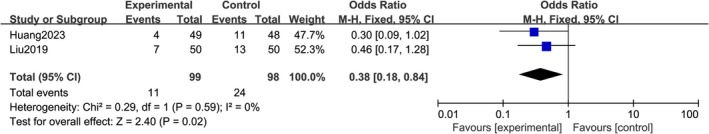
Forest of adverse reactions.

Two studies reported adverse reactions, with no statistical heterogeneity between each study (*I* ^2^ = 0%, *p =* 0.59), using a fixed effects model. The meta‐analysis results showed a statistically significant difference in adverse reactions between the two groups [*RR =* 0.38; 95% CI (0.18, 0.84); *p =* 0.02]. The results indicate that injecting botulinum toxin A around the wound can reduce adverse reactions, as shown in Figure 9.

### 3.4 Sequential Analysis Results of Experiments

All 9 research data were included in the sequential analysis of the Visual Analog Scale (VAS) trial. Using a random effects model, O'Brien Fleming was used to calculate the one‐sided *Z*‐score threshold, with a set of 5% type I errors and 20% type II errors for each side. The positive rate of the control group was calculated based on the included studies, and the relative risk reduction was estimated based on studies with low bias risk. The information content was estimated based on available statistical information. The results showed that the *Z*‐value curve of MACE crossed the threshold of sequential analysis in the experiment, indicating that the observed effect of current information is conclusive, and local injection of botulinum toxin A can significantly improve scar quality.

#### 3.4.1 Publication Bias

**FIGURE 10:**
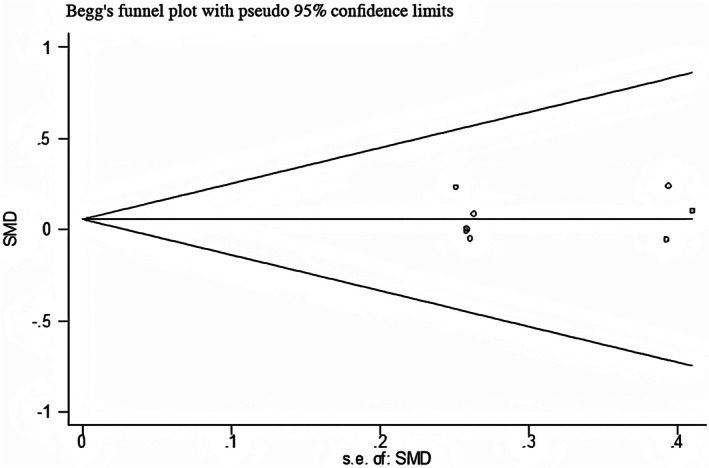
Efficiency Begg's test.

**FIGURE 11:**
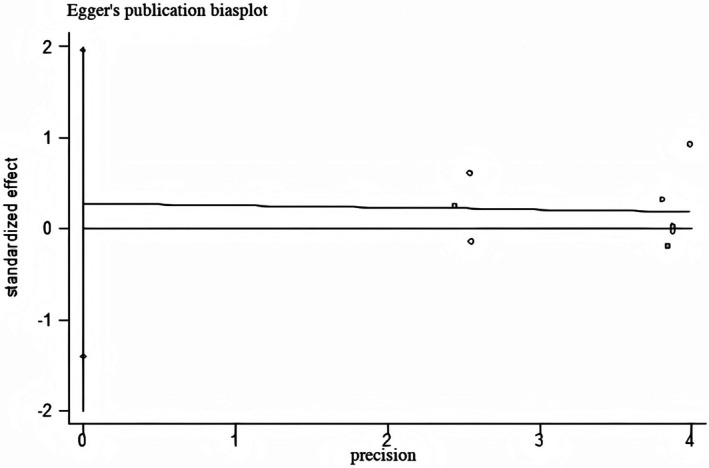
Efficiency Egger's test.

This study conducted Begg's test and Egger's test on the Visual Analog Scale (VAS) to determine the overall publication bias of the article. The specific results are shown in Figures 10 and 11. Begg's test showed *p =* 0.83 > 0.05, and Egger's test showed *p =* 0.39 > 0.05, both indicating no publication bias.

### 3.5 Sensitivity Analysis

**FIGURE 12:**
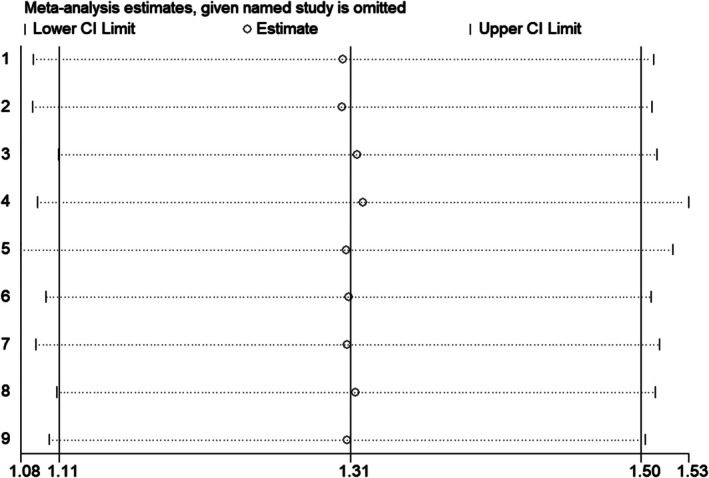
Sensitivity analysis forest map included in the literature.

This study used the metaninf module of STATA12.0 software to detect the impact of each individual study on the overall combined effect. As shown in Figure 12, after excluding each study one by one, the combined effect of the remaining studies did not show significant changes, indicating that the stability of the results in this study is relatively good.

## 4 Discussion

This meta‐analysis included 644 patients from 12 randomized controlled trials (RCTs). The results demonstrated that local injection of botulinum toxin type A (BTX‐A) significantly improved multiple scar outcomes compared to the control group. Specifically, BTX‐A treatment reduced the Vancouver Scar Scale (VSS) score by an average of 3.1 points, decreased scar width by 1.3 mm, and lowered the pain/itch visual analog scale (VAS) score by 18 mm. Furthermore, patient satisfaction increased from 64% to 87% in the BTX‐A group. Trial sequential analysis confirmed that the cumulative evidence surpassed the required information size, providing conclusive evidence for the efficacy of BTX‐A in preventing pathological scar formation in the maxillofacial and cervical regions.

The pathogenesis of keloids remains incompletely understood. Their high incidence in high‐tension areas such as the chest, shoulders, and back suggests that excessive mechanical tension is a key predisposing factor [31]. Supporting this, studies have shown that reducing skin tension can improve scar outcomes and exert a preventive effect [32]. It is hypothesized that reducing muscle tension via BTX‐A may help re‐establish an equilibrium between fibroblast apoptosis and proliferation, thereby effectively preventing and treating scars [33].

Botulinum toxin type A is a potent neurotoxin that acts on cholinergic nerve endings to inhibit muscle fiber contraction and reduce tension at the margins of healing wounds [34]. Additionally, it may immobilize fibroblasts in the G0 and G1 phases of the cell cycle (non‐proliferative state) [35] and downregulate the expression of transforming growth factor‐beta 1 (TGF‐β1). By modulating TGF‐β1 signaling, BTX‐A may directly regulate fibroblast activity, altering apoptosis, migration, and fibrotic processes, ultimately reducing scar formation. A previous large‐sample meta‐analysis of high‐quality studies indicated that intralesional injection of BTX‐A was more effective in treating pathological scars than triamcinolone acetonide alone or placebo and significantly reduced post‐injection pain [36].

All studies included in this analysis were RCTs, thus minimizing selection bias. However, several limitations should be acknowledged. First, potential systematic errors may arise from variations in clinical patient characteristics. Second, sources of clinical heterogeneity include differences in surgical incision location and length, variations in the timing and dosage of BTX‐A injection, and disparities in whether the patient was undergoing primary surgery or revision surgery, as well as follow‐up duration. These factors are likely the primary contributors to the observed heterogeneity. In this study, high heterogeneity (*I* ^2^ = 100%) was observed only for the visual analog scale (VAS) outcome measure, while the heterogeneity for other important outcome measures was low (*I* ^2^ < 50%), indicating good consistency. This suggests that the heterogeneity may primarily stem from the measurement or reporting methods of the specific VAS score, rather than a general inconsistency in the effects of the intervention across all aspects. In this context, extensive exploratory subgroup analysis based solely on this single heterogeneous outcome measure may lead to overinterpretation of coincidental findings and has limited reference value for forming overall conclusions. Therefore, no subgroup analysis was conducted.

The pooled results indicated a significantly higher patient satisfaction rate in the BTX‐A group than in the control group. This improvement may be attributed to the ability of BTX‐A to inhibit the release of substance P and other neuropeptides, thereby reducing symptoms of erythema and pruritus in scar tissue. Although some studies have reported on the analgesic properties of BTX‐A, its precise mechanism remains unclear. Furthermore, Filipovic et al. reported that BTX‐A injection can persistently alleviate wound pruritus and pain, improving patient comfort [37].

This study employed meta‐analysis and trial sequential analysis (TSA) to reassess post‐treatment VSS and VAS scores, further elucidating the value of BTX‐A in scar management. Unlike traditional meta‐analyses, which may inflate random errors and increase the risk of false‐positive results due to multiple testing, TSA effectively mitigates these limitations. Our findings revealed that the BTX‐A group achieved significantly lower VSS and VAS scores than the control group (*p* < 0.05), indicating that BTX‐A injection can effectively reduce scar severity and alleviate pain. The TSA results confirmed the conclusiveness of these findings, which are consistent with large‐sample RCTs published by other scholars.

Normally, external stimuli excite C‐type neurons, leading to the release of sensory neuropeptides (e.g., substance P) that transmit impulses to the central nervous system, resulting in pain sensation. Under pathological conditions, the excitation threshold of C‐type neurons is lowered, meaning even mild stimulation can trigger substance P release, causing sensations of pain and pruritus. Substance P promotes degranulation of mast cells and platelets, releasing histamine and other mediators that stimulate nerve endings, producing pain, itching, and discomfort. The inflammatory response during hypertrophic scar repair releases various mediators, including platelet‐activating factor, serotonin, histamine, proteases, kallikrein, substance P, and bradykinin. These substances act on C‐type nerve endings, leading to persistent pain and pruritus that significantly impact the patient's quality of life [38, 39].

Scar width, a key objective indicator of scar hypertrophy, can be directly observed and measured. Our analysis found a statistically significant reduction in scar width in the BTX‐A group compared to the control group. The potential mechanisms include: (1) BTX‐A acts on peripheral motor nerve endings, inducing muscle relaxation, reducing wound tension, and consequently reducing collagen deposition and scar hypertrophy; (2) BTX‐A inhibits the proliferation and differentiation of myofibroblasts within scars, suppressing scar contraction and proliferation; (3) BTX‐A inhibits fibroblast proliferation in scar tissue, reducing collagen matrix secretion and promoting scar tissue atrophy [40].

This study found that the recurrence rate and incidence of adverse reactions were significantly lower in the BTX‐A group than in the control group (both *p* < 0.05), indicating that BTX‐A can inhibit scar hyperplasia, consistent with previous reports [15, 16, 17, 18, 19, 20]. The potential mechanism involves the inhibition of fibroblast proliferation. Studies have identified fibroblasts, macrophages, neutrophils, and myofibroblasts as key cells driving scar hyperplasia [41]. Among these, fibroblasts are paramount, as they also secrete cytokines promoting fibrotic tissue formation. BTX‐A may inhibit fibroblast proliferation and collagen synthesis by modulating the expression of TGF‐β1 and matrix metalloproteinases (MMPs) such as MMP‐1 and MMP‐2 [22, 42].

Compared with previous systematic reviews, the innovation of this study is mainly reflected in the following three aspects: (1) It is the first time to introduce Trial Sequential Analysis (TSA) in the meta‐analysis of BTX‐A for preventing facial scars, confirming the robustness of the conclusions and the sufficiency of the sample size, thus overcoming the risk of false positives that may arise in traditional meta‐analysis; (2) It specifically focuses on facial wounds and post‐plastic surgery scars, starting from the unique mechanical tension mechanism of this area, and deeply explores the pathological mechanism of BTX‐A in reducing fibrosis by inhibiting the activity of facial muscles, thus compensating for the lack of generalization in previous research locations; (3) It integrates multiple indicators such as patient satisfaction, VSS score, VAS score, scar width, and recurrence rate to comprehensively evaluate the clinical efficacy of BTX‐A, providing a richer evidence‐based basis for early intervention of facial scars.

The therapeutic effect of preventing hypertrophic scars in the maxillofacial and neck regions. However, after conducting this analysis, there are still some shortcomings in the results of this study. ① The age differences between the populations in each study were not taken into account. The patients included in this study ranged in age from 3 months to 70 years old. As mentioned earlier, hypertrophic scars mostly occur in adolescents, so the degree of scar hyperplasia may vary depending on the age group, which cannot avoid clinical heterogeneity; ② The basic characteristics of patients vary among different studies, and the diagnostic and safety evaluation criteria are also not unified. These factors may have a certain impact on the results; ③ Due to the relatively small sample sizes among various studies, the number of included literature and evidence is limited, and the scientific and experimental validity of the results needs further exploration, research, and supplementation; ④ Most of the included studies in this research originated from Chinese databases. While this may provide a more comprehensive reflection of research on the Chinese population, it could also introduce regional and linguistic biases, thereby limiting the generalizability of conclusions to non‐Chinese or non‐Asian populations. ⑤ This study failed to delve into potential sources of heterogeneity through subgroup analysis. This is due to the limited number of included studies, as performing subgroup analysis would further divide the sample size, leading to insufficient statistical power. Additionally, most of the original studies did not fully report key variable data, increasing the risk of false positive conclusions due to multiple comparisons.

In summary, local injection of BTX‐A demonstrates significant efficacy in both the treatment and prevention of hypertrophic scars, with no obvious side effects. Future research needs to further clarify the relevant cellular signaling pathways of BTX‐A, explore the mechanisms of BTX‐A in treating scars, commit to conducting pre‐specified subgroup analyses, and require higher quality, larger sample sizes, and longer follow‐up clinical randomized controlled trials to clarify the clinical efficacy of treatment. Research comparing BTX‐A with other scar prevention methods can be carried out, or the long‐term effects of BTX‐A can be explored in order to derive more reliable theories to guide clinical work and reduce patients' disease and psychological burden.

## Author Contributions

We declare that all the listed authors have participated actively in the study and all meet the requirements of authorship. J.L. prepared the paper, managed the literature searches and data analysis, Y.C. undertook the statistical analysis, H.X. edited the paper, Y.W. designed the study, contributed to the correspondence and paper revision. All authors reviewed the manuscript.

## Ethics Statement

All analyses were based on previously published studies, thus no ethical approval and patient consent are required.

## Consent

The authors have nothing to report.

## Conflicts of Interest

The authors declare no conflicts of interest.

## Data Availability

The datasets used or analyzed during the current study are available from the corresponding author on reasonable request.
